# Framework for Implementing Individualised Dosing of Anti-Cancer Drugs in Routine Care: Overcoming the Logistical Challenges

**DOI:** 10.3390/cancers15133293

**Published:** 2023-06-22

**Authors:** Jason van Leuven, Simon Evans, Ganessan Kichenadasse, Neeltje Steeghs, Billie Bonevski, Gerd Mikus, Madelé van Dyk

**Affiliations:** 1 College of Medicine and Public Health, Flinders University, Adelaide 5042, Australia; 2 Medical Oncology, Flinders Medical Centre, Adelaide 5042, Australia; 3 Implementation Science Unit, Department for Health and Wellbeing, Adelaide 5042, Australia; 4 Antoni van Leeuwenhoek Netherlands Cancer Institute, 1066 CX Amsterdam, The Netherlands; 5 Department of Medical Oncology, Netherlands Cancer Institute, 1066 CX Amsterdam, The Netherlands; 6 Department of Clinical Pharmacology and Pharmacoepidemiology, University Hospital Heidelberg, 69120 Heidelberg, Germany

**Keywords:** implementation science, individualised dosing, personalised medicine, therapeutic drug monitoring, oral anti-cancer drugs

## Abstract

**Simple Summary:**

Many anti-cancer drugs are prescribed at a ‘one-size fits all’ starting dose, without considering individual differences. This is a problem for many cancer patients as this fixed dose might be too much for an individual which could lead to adverse effects and toxicity. Similarly, the fixed-dose might not be enough for some patients which could lead to therapeutic failure. Achieving the right amount of anti-cancer drugs in the body is critical, however, currently, the anti-cancer drug concentrations are not monitored in routine care. Our group used our extensive experience, existing published frameworks and recommendations to establish our own framework to implement a program that enables individualized dosing of anti-cancer drugs in routine care. This paper describes this framework with a simple 6-step process that can be utilised for the implementation of individualised dosing programs for anti-cancer drugs.

**Abstract:**

Precision medicine in oncology involves identifying the ‘right drug’, at the ‘right dose’, for the right person. Currently, many orally administered anti-cancer drugs, particularly kinase inhibitors (KIs), are prescribed at a standard fixed dose. Identifying the right dose remains one of the biggest challenges to optimal patient care. Recently the Precision Dosing Group established the Accurate Dosing of Anti-cancer Patient-centred Therapies (ADAPT) Program to address individualised dosing; thus, use existing anti-cancer drugs more safely and efficiently. In this paper, we outline our framework, based on the Medical Research Council (MRC) framework, with a simple 6-step process and strategies which have led to the successful implementation of the ADAPT program in South Australia. Implementation strategies in our 6-step process involve: (1) Evaluate the evidence and identify the cancer drugs: Literature review, shadowing other experts, establishing academic partnerships, adaptability/flexibility; (2) Establishment of analytical equipment for drug assays for clinical purposes: assessment for readiness, accreditation, feasibility, obtaining formal commitments, quality assurance to all stakeholders; (3) Clinical preparation and education: educational material, conducted educational meetings, involve opinion leaders, use of mass media, promote network weaving, conduct ongoing training; (4) Blood collection, sample preparation and analyses: goods received procedures, critical control points (transport time); (5) Interpret and release results with recommendations: facilitate the relay of clinical data to providers; (6) Clinical application: providing ongoing consultation, identify early adopters, identify, and prepare champions. These strategies were selected from the 73 implementation strategies outlined in the Expert Recommendations for Implementing Change (ERIC) study. The ADAPT program currently provides routine plasma concentrations for patients on several orally administered drugs in South Australia and is currently in its evaluation phase soon to be published. Our newly established framework could provide great potential and opportunities to advance individualised dosing of oral anti-cancer drugs in routine clinical care.

## 1. Introduction

Optimal precision medicine in oncology involves identifying the ‘right drug’, at the ‘right dose’, for the right person. Identifying the right dose remains the biggest challenge to optimal patient care. Currently, many orally administered anti-cancer drugs, particularly the kinase inhibitors (KIs), are prescribed especially at the time of treatment initiation with a standard fixed dose as per the approved drug label [1,2,3]. The fixed doses are often determined in early phase clinical trials using relatively small numbers of patients. These clinical trials exclude patients with characteristics typically seen in the community (e.g., those with poor performance status, the elderly, those on concomitant medications, co-morbidities, diverse racial/ethnic composition) [4]. It is evident that, even in the ‘homogenous’ clinical trial populations, a wide variability of drug exposure measured as various pharmacokinetic (PK) parameters (such as the minimum concentration, C_min_; maximum concentration, C_max_; area under the concentration–time curve, AUC) is observed [5,6]. Drug exposure is often related to clinical outcome (cancer response and toxicities) which consequently also varies significantly. As the clinical trial patient groups do not reflect the general population, when a fixed dose of drugs derived from clinical trial cohorts is applied to real-world patients, a much wider variability in cancer response/toxicity is observed [3,5]. Indeed, when a fixed dose is applied to the general population, significant variability in drug exposure has been reported (coefficient of variation, CV% 19 to >105 between patients) [3,7,8].

**Figure 1:**
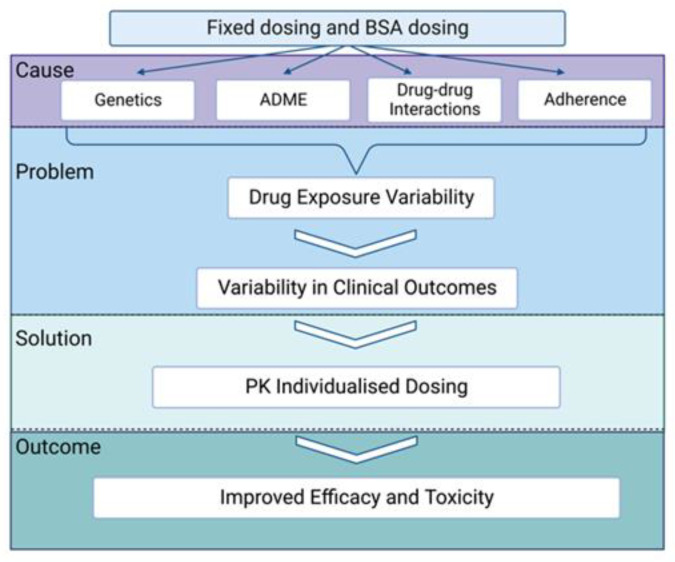
The cause, solution, and outcome of pharmacokinetic variability when a fixed dose is used, adapted from de Wit, 2015 [1].

Several patient, drug and disease-related factors can contribute to the variability. Some of the variability is explained by genetics and genomics (patient and tumour); pharmacokinetics (absorption, distribution, metabolism, and excretion (ADME)) of the drug; drug–drug interactions; and adherence to the drug [4,8] (Figure 1). This need to address variability in drug concentrations and response between individuals has also been recognised by the Food and Drug Administration (FDA); hence, the FDA’s Oncology Centre of Excellence has launched Project Optimus which focusses on dose selection of drugs during oncology drug development [9].

One method of individualised dosing is using therapeutic drug monitoring (TDM) with an adjustment dosing strategy; for this, plasma or serum are typically used to measure the drug concentration and if it is too low or too high, an adjustment can be made to optimise the dose for each patient. TDM is considered beneficial for drugs that typically are used long term, have no easy to measure biomarker to evaluate the drug effect, exhibits an exposure-response relationship with a narrow therapeutic index, have a well-defined target concentration, as well as have a dose modification approach [8,10]. Applying the TDM approach to anti-cancer drugs which have well-defined targets, has shown to be beneficial in improving clinical outcomes [3,11,12]. Previous research supports the routine use of TDM for many kinase inhibitors (KIs), such as everolimus (target concentration of ≥10 µg/L) [13,14,15,16], imatinib (≥1100 µg/L) [17,18,19,20,21,22,23], sunitinib (≥37.5 µg/L with continuous dosing, ≥50 µg/L with intermittent dosing) [24,25], pazopanib (≥20,000 µg/L) [26,27,28,29,30,31,32] with several others, described in our recently published review [3]. Despite evidence showing a progression free survival (PFS) or overall survival (OS) benefit, plasma concentration guided dosing of anti-cancer drugs has not been translated into clinical practice. Among various reasons, lack of access to technical platforms (analytical methodology and infrastructure for measuring drug concentrations) and clinician awareness (clinical evidence and recommended action) were the dominant challenges for failed translation and implementation [3,8,11,12].

We (a team of multidisciplinary researchers including medical oncologists, pharmacologists, pharmacists, and consumers) have established the Accurate Dosing of Anti-cancer Patient-centred Therapies (ADAPT) Program to address the challenges (lack of knowledge and infrastructure) in implementing individualised dosing and thus use existing anti-cancer drugs more safely and efficiently. The first wave of drugs on the program were selected from our review which described high level available evidence in support of individualised dosing [3]; in the current instance imatinib, sunitinib, pazopanib, erlotinib, gefitinib, and sorafenib were selected and several more drugs with this level of evidence are currently being added (e.g., abiraterone, axitinib, crizotinib and nilotinib). It should be noted that the cancer therapies are rapidly evolving, the program is continuously changing and being updated with the next wave of drugs. The program has a multi-faceted approach consisting of a research, feasibility, implementation, and evaluation phases. This paper provides a framework for establishing a platform for individualised dosing of cancer drugs using the ADAPT program. Our outlined framework, six-step process and strategies gives others a starting-point and the opportunity to tailor similar programs to their clinical need and setting.

## 2. General Framework and Six-Step Process for Establishing Individualised Dosing Programs

**Figure 2:**
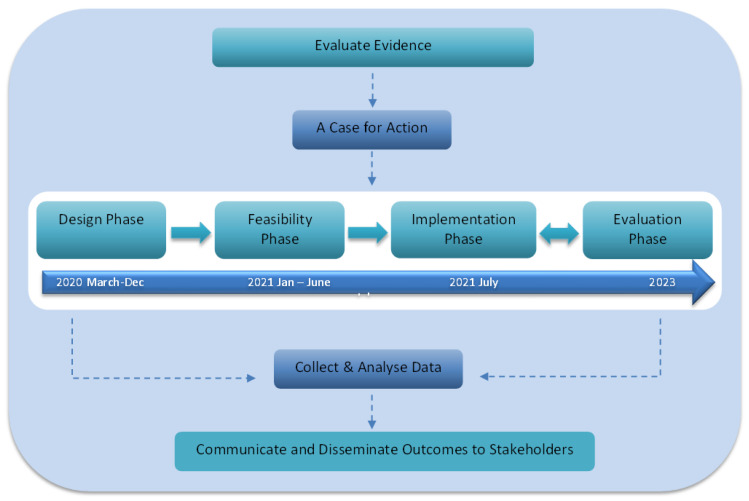
The general framework for establishing programs for individualized dosing of cancer drugs.

As initial steps for implementing individualised dosing of anti-cancer drugs in routine clinical practice, a case for action based on the available evidence and clinical need must be generated and evaluated (Figure 2) [33]. Translation into clinical practice is more likely to occur with a strong case for action; however, there are many different factors that can affect the uptake of a clinical practice. The need for our individualised dosing program arose due to the rapidly evolving evidence and the unsatisfactory nature of cancer therapies with fixed dosing of oral kinase inhibitors. The ADAPT program was based on the Medical Research Council (MRC) framework for developing and evaluating complex interventions [34,35,36,37,38]. We utilised the four key phases: Development/Design, Feasibility/Piloting, Implementation and Evaluation (Figure 2).

**Figure 3:**
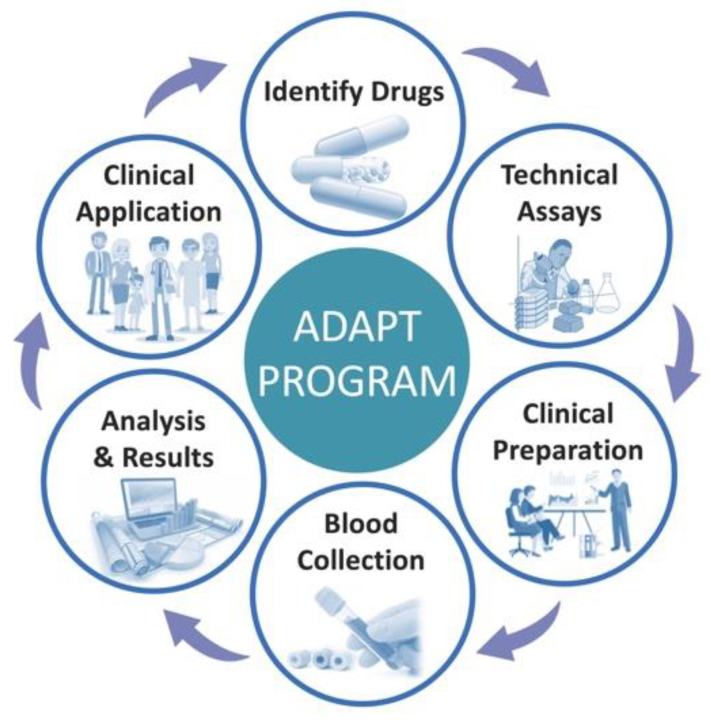
The six-step process in establishing the ADAPT program.

The development/design phase largely focused on the establishment of the operational and logistical processes of the program through a six-step process (Figure 3) [33,34]. This six-step process included an evaluation of evidence for the identification of anti-cancer drugs; setting up of technical assays; clinical preparation (e.g., clinician education, patient selection); blood collection; blood sample analyses, release of results with dosing recommendations; and clinical application of recommendations. Each of these steps required the appropriate use of implementation strategies which is outlined below in Section 2.

This design phase was followed by a feasibility phase during which a pilot study of the ADAPT program and the six-step process was conducted. Following this, the implementation phase was launched in parallel with a continuous evaluation phase where every component of the program was assessed with outcome measures. The six-step process is described below: 

### 2.1. Step 1: Evaluate the Evidence and Identify the Cancer Drugs

To identify potential anti-cancer drugs for individualised dosing, a literature review was conducted. An existing systematic review summarising available evidence for oral anti-cancer drugs published in collaboration with leading experts such as the Dutch Pharmacology Oncology Group (DPOG), PharMetrX Group, various others and our PD Group was also used as a valuable starting point [3]. We utilised a previously described criteria by the DPOG for identifying potential drug candidates for individualised dosing [10]. Shadowing other experts who are currently individualising doses of anti-cancer drugs was a beneficial strategy; thus, it may be valuable to establish academic partnerships with other institutes and universities to share resources and expertise [39,40]. Oral anti-cancer drugs are rapidly evolving, and first line therapies are often replaced by newer generation drugs [3]. Sufficient evidence may not yet be available for such newer anti-cancer drugs; therefore, we recommend establishing a research and development (RandD) division to run in parallel with the individualised dosing program. The RandD division enables the continuous evaluation of new anti-cancer drugs, while generating further evidence for existing anti-cancer drugs. To carry out the RandD, we recommend following steps 1–5, as the studies are observational and do not include any clinical application (step 6). To maintain the viability of the individualised dosing program adaptability and flexibility needs to be adopted, allowing for the rapidly changing nature of clinical care. For all research studies carried out, appropriate ethics and governance approvals need to be sought.

### 2.2. Step 2: Establishment of Analytical Equipment for Drug Assays for Clinical Purpose

Establishing the infrastructure with the appropriate equipment and facilities, staffed by experienced and qualified laboratory personnel, was vital in establishing an analytical laboratory [41]. It is important that before commencing analytical work an assessment for readiness is performed and possible facilitators and barriers identified [39,40]; furthermore, the development and validation of assays must be undertaken to facilitate drug quantification. Liquid chromatography-mass spectrometry (LC-MS) assays was our preferred method over other bioanalytical methods, due to its high sensitivity and the ability to accurately quantify virtually any compound [42]; instrument configuration, compound-specific and chromatography settings need to be optimised. The FDA, Therapeutic Goods Administration (TGA) and European Medicines Agency (EMA) have set guidelines for the validation of bioanalytical assays for drug quantification which have to be strictly followed [43]. Thereafter, the appropriate accreditation must be obtained by authorities; for example, in Australia, laboratory accreditation (compliance and governance) is gained through the National Association of Testing Authorities (NATA). Consequently, the feasibility of conducting drug quantification assays need to be tested to ensure patient samples can be analysed appropriately within the specified timeframes. In the case of outsourcing bioanalytical assays, formal commitments should be obtained via a written service agreement with the laboratory to ensure high-quality measurements within fixed timeframes [39,40]. Following this, implementation can be launched to support sample analyses (step 5) and should be continuously evaluated for assay accuracy and performance. This ensures that experiments are carried out with the highest precision and reliability, giving quality assurance to all stakeholders. Alternatively, a coalition can be built with other national and international laboratories to develop and organise quality monitoring systems [39,40], if accreditation is not required or possible; regular cross-validation between laboratories with in-house assays can be performed, as part of a quality assurance program (QAP), which can be established by the laboratories themselves or commercial QAPs can be used, if available.

### 2.3. Step 3: Clinical Preparation and Education

For any clinical translation and implementation to occur, stakeholders need to be informed on the various processes involved regarding the dose individualisation of anti-cancer drugs [44]. A literature review was conducted to select and plan the most suitable strategies to initiate the clinical preparation and education process for all stakeholders involved [45,46,47,48,49,50,51,52,53,54,55,56,57,58]. To achieve this, we developed educational material and conducted educational meetings, involved opinion leaders, and audited and incorporated feedback to monitor the successful use of these strategies. The use of mass media was an effective strategy to promote the ADAPT program and educate the general population, patients/consumers, and their carers; for example, patient information pamphlets and media releases such as local and national television news or radio interviews. It is strongly encouraged to promote network weaving (e.g., face-to-face presence within the clinic and sharing data through conferences and publication) [39,40], and build on high-quality relationships between oncologists, patients, pharmacists, bioanalytical laboratory personnel, and other stakeholders, especially when developing the educational material. It is important to engage with these various stakeholders to ensure the development of educational material is appropriate for the target audience. As a part of implementation, successful education can be measured by the uptake of the program during ongoing evaluations. Conducting ongoing training (e.g., local, state-wide, or national workshops) at regular timepoints while ensuring training is accessible to all stakeholders is an essential ingredient for success.

### 2.4. Step 4: Blood Collection, Sample Preparation and Analyses

For an individualised dosing program using plasma concentration, either in routine care or a research setting, blood collection from the patient is required to be taken by a qualified phlebotomist; this can be a student, research assistant or healthcare professional. Blood collection can be performed at either a registered blood collection centre or an in-house facility supplied with the necessary consumables, equipment, and storage conditions. For the drugs on the ADAPT program (and most oral anti-cancer drugs) a 2–5 mL blood sample should be taken at a trough concentration (also known as ‘minimum concentration or C_min_’, which needs to be taken within 0–60 min before the next dose) at steady state (two to three weeks after treatment initiation and once every three months thereafter for the duration of the treatment. A trough sample at steady state should also be repeated after a dose change. The samples then need to be processed, prepared for analyses, and stored in an appropriate environment. As part of the design and feasibility phases various strategies can be trialled; however, prior to the implementation phase the most efficient and user-friendly blood sample collection procedure should be adopted. The ADAPT Program is implemented at state level; therefore, we focused on maximising patients’ accessibility and convenience by utilising established standard routine care for blood collection, allowing patients from any location to access the service. in transporting the blood samples from the collection facility to the laboratory, particular consideration needs to be taken regarding temperature and exposure to light. The laboratory will need to have a goods received procedure, where critical control points are monitored (e.g., temperature). Once processed, samples will need to be placed in the appropriate refrigerated storage until they are ready to be analysed. Samples must be prepared and analysed by trained and experienced laboratory personnel, who can confidently perform assays on High Performance Liquid Chromatography (HPLC), or LC-MS.

### 2.5. Step 5: Interpret and Release Results with Recommendations

Once the sample analyses have occurred at the selected laboratory, the result must be released onto a platform accessible to the intended healthcare professional such as the treating clinician or pharmacist. We chose to facilitate the relay of clinical data to providers in real-time; this can range anywhere from email, hard copy or electronic medical records. If hard copies are used it needs to be faxed and emailed to the pharmacist to be included in the patients’ medical file ready for the treating clinician or directly to the treating clinician. The results also need to be sent to the specialist team responsible for the interpretation of the result such as the assigned clinical pharmacologists or pharmacists, this can also be achieved via email, hard copy or shared secure password protected electronic folders within the health networks’ system. Prior to the commencement of any individualised dosing program, clear evidence-based dosing protocols need to be established; we utilised a previously published protocol established by the DPOG [12]. Individualised dosing results needs to be interpreted by trained staff and clinical pharmacologists. After interpretation, dose recommendations need to be communicated to the treating clinicians via the selected communication pathways as mentioned above. For both the release of the result and the interpretation along with dosing recommendation, various strategies should be trialled until the most efficient and preferred pathway is chosen.

### 2.6. Step 6: Clinical Application

For any application of an intervention (implementation) to occur, a change in behaviour is required from the intended treating clinician [49]. This is typically directly related to the quality of the education and training strategies utilised for clinical preparation (Section 2.3, Step 3). Once the treating oncologist/haematologist receives the result and dosing recommendations, it is then up to the clinician to apply the recommendations. However, providing ongoing consultation to the clinician by the clinical pharmacologist is an important strategy which can help to identify early adopters [39,44]. It is here we can identify and prepare champions of individualised dosing who help assist in marketing and overcome resistance to the individualised dosing program [39,44]. Champions can also assist in gaining some understanding into the behavioural barriers as to why some clinicians may be resistant or slow to adopt an individualised dosing program [45,46,49,51]. As part of implementation of a newly introduced intervention/program, scheduled auditing should occur at regular intervals of 6 or 12 months depending on the complexity of the healthcare delivery and unexpected challenges encountered. The time taken from drug concentration request to blood sample collection, to blood sample arrival and processing, to the release of the result, to dosing recommendation, and application of dose recommendation should also be documented as part of the evaluation phase to ensure continuous improvement. The success of the clinical application is measured by the percentage of followed dose recommendations (number of dose adjustments made/number of dose adjustments recommended × 100). Similarly, the dose recommendations should also be audited by reviewing each patient’s analysed plasma concentration result, and whether the appropriate dose adjustments were made for each of the drugs. During implementation, based on the auditing results, various tools such as questionnaires to better understand clinicians’ current knowledge and/or reasons for not taking up the program can be developed to encourage the change in clinician behaviour [49,50], and as part of the evaluation phase.

## 3. Continuous Evaluation: Identification of Facilitators and Barriers

An important step in translating any clinical knowledge into a clinical program is to continuously audit and provide feedback; this approach not only keeps all stakeholders engaged but advances the quality of the program. This continuous improvement approach seeks to leverage facilitators and overcome barriers. Additionally, incentives can be effective, where incentivising change can facilitate a new behaviour, which can coincide with reminder systems and other efficiencies. Facilitators and barriers can be found at the individual, interpersonal, organisational, community and public policy level—and can be either physical or psychological. Once these facilitators and barriers have been identified the literature must be reviewed to see if these have been reported and addressed in similar implementation settings. It is important to always search for existing guidelines in your area in peer-reviewed literature. Alternatively, guidelines for translation and implementation for different medicines or initiatives/programs can be used.

## 4. Discussion

Therapeutic drug monitoring and individualised dosing of cancer drugs is not novel. It is a concept that has been recognised for more than 30 years now, and even longer for other drug classes such as antimicrobials. However, the implementation of TDM and individualised dosing has failed due to practical challenges, lack of guidelines and uncertainty of whose role and responsibility it is to implement and operate [3,41,59].

This paper provides guidelines on how to implement an individualised dosing program of anti-cancer drugs in routine care. At the core of this framework is a 6-step process which summarises the operational and logistical steps required to establish and implement an individualised dosing program.

It is important to start by reviewing and evaluating the literature to identify the appropriate anti-cancer drug candidates suitable for individualised dosing; however, existing eligibility criteria can be used [7,11,12,59]. Additionally, stakeholders, such as prescribers of the selected drugs (oncologists/haematologists) and consumers, can also be approached to inform your decision; Mueller et al. (2021) have also categorised the strength of evidence for oral anti-cancer drugs eligible for individualised dosing [3]. We emphasise the importance of establishing a bioanalytical laboratory that is both managed by expert analytical chemists and accredited by the appropriate regulatory authorities in order to perform drug quantification assays for clinical purposes.

Education plays a key role in clinical preparation and the educational material needs to be developed, customised, and directed to the specific stakeholder audiences, such as oncologists, pharmacists, nurses, laboratory personnel or consumers [60,61]. We highly recommend involving opinion leaders or champions, such as oncologists/haematologists and clinical pharmacologists, to help deliver the educational material; this helps to increase acceptance of the individualised dosing program in routine care [62,63,64].

Blood collection needs to be undertaken by the most user-friendly procedure available; hence, we highlight the advantages of outsourcing to an establish and accredited blood collection service that has all the necessary standard operating procedures in place [65]. The sample preparation and analyses are dependent on the successful establishment of the analytical equipment for drug assays for clinical purpose. Results need to be made available and integrated into the healthcare system in a way that is familiar or recognisable by the treating oncologist/haematologist or other healthcare professionals involved. Results also need to be sent to the clinical pharmacology team to be interpreted, which requires additional responsibility; therefore, clear expectations and allocated time need to be arranged to meet the clinical application of the dose recommendation (if any) in a timely manner.

Probably the most important part of the process is the implementation of the recommendations (dose adaptation) by the treating oncologist/haematologist. This only occurs when you provide ongoing consultation to the treating clinician. With the right support you can identify and prepare these early adopters as champions, allowing them to tell others about their own experience to the precision dosing program/platform. It is important to conduct periodical audits to ensure the clinicians are applying the dose recommendations.

## 5. Conclusions

Implementation of individualised dosing of cancer drugs in routine care is highly complex and difficult to achieve due to the various stakeholders and multi-faceted operations involved. It needs to be acknowledged that for any implementation of an individualised dosing program for cancer drugs, a multi-disciplinary approach has to be undertaken; for this, appropriate guidelines and a framework specific to the drug class and patient population are necessary. Based on our previous clinical experience we have provided appropriate guidelines and a framework with a 6-step process that can be utilised for the implementation of individualised dosing programs for cancer drugs. In this 6-step process, we outline implementation strategies necessary for each of the specific day-to-day and routine processes involved. Our framework and the 6-step process have led to the successful establishment of the ADAPT program within routine care and are currently in their implementation and evaluation phase; preliminary results are presently being collated to be published in early 2023.
